# Elucidation of inorganic nitrogen utilization characteristics in *Coix lacryma-jobi* L. seedlings under variable ammonium/nitrate ratios via the ^15^N tracer technique

**DOI:** 10.1186/s12870-026-08825-y

**Published:** 2026-04-28

**Authors:** Yue Su, Kaiyan Zhang, Yanyou Wu

**Affiliations:** 1 Engineering Technology Research Center for Protection and Detection of Germplasm Resources of Karst-Adaptable Crops, Guizhou Vocational College of Agriculture, Qingzhen, 551400 China; 2 School of Karst Science, Guizhou Normal University/State Engineering Technology Institute for Karst Desertification Control, Guiyang, 550025 China; 3 State Key Laboratory of Environmental Geochemistry, Institute of Geochemistry, Chinese Academy of Sciences, Guiyang, 550081 China

**Keywords:** Ammonium, Nitrate, ^15^N labeling technique, Nitrogen accumulation amount, Nitrogen utilization efficiency

## Abstract

The coordination between nitrogen assimilation and carbon fixation is a key determinant of crop productivity. However, for *Coix lacryma-jobi* L.—an underutilized cereal with high nutritional and medicinal value—the interplay between nitrogen source and photosynthetic performance remains unknown, limiting the development of precision cultivation practices. In this study, ¹⁵N-labeled ammonium chloride (¹⁵NH₄Cl, 10 atom% ^15^N) was employed as the tracer source for ammonium, and unlabeled sodium nitrate (NaNO_3_) with natural ¹⁵N abundance was used as the nitrate. Four ammonium/nitrate ratios (10/90, 30/70, 50/50, 70/30) were established under hydroponic conditions. On the basis of the ^15^N labeling technique, the nitrogen accumulation amount derived from ammonium and nitrate utilization could be quantified for *Coix* seedlings grown under different ammonium/nitrate ratios. Under the 50/50 ratio of ammonium/nitrate treatment, the *Coix* seedlings achieved the maximum carbon accumulation, which may be attributed to the synergistic effect of minimized energy expenditure for nitrogen assimilation (approximately 60% ammonium utilization) and a concomitant boost in photosynthetic capacity. However, excessive ammonium assimilation (> 70%) under the highest ammonium/nitrate ratio significantly reduced both photosynthetic capacity and the utilization efficiency of ammonium and nitrate in *Coix* seedlings. The high reductant demand from intense nitrate assimilation (> 85%) under the lowest ammonium/nitrate ratio limited photosynthetic carbon fixation, thereby reducing total carbon accumulation in *Coix* seedlings. The integration of the photosynthetic performance with the characteristics of inorganic nitrogen utilization contributes to revealing nitrogen metabolism efficiency and its coordination with carbon assimilation. Furthermore, quantifying the nitrogen accumulation amount derived from ammonium utilization and nitrate utilization offers insights into precision nitrogen management for *Coix* seedlings, contributing to sustainable agricultural practices.

**Supplementary Information:**

The online version contains supplementary material available at 10.1186/s12870-026-08825-y.

## Background

Nitrogen is a vital element for plant growth. Its concentration and form significantly influence the growth and development of plants. In agricultural soil, nitrate and ammonium constitute the primary forms of inorganic nitrogen. The mean concentrations of aerobic agricultural soil nitrate and ammonium have been reported to be 4.5 and 0.77 mM, respectively [1]. In general, nitrate is the main nitrogen source utilized by most plants under aerobic soil conditions [2]. Plants usually show a strong flexibility in the uptake of inorganic nitrogen to maximize the utilization of available nitrogen in soils [3–5]. Given that nitrate is easily leached from soils during rainfall [6, 7] and its assimilation requires more energy compared to ammonium [8, 9], optimizing the ammonium/nitrate ratio and reducing the supply of inorganic nitrogen contributes to improving nitrogen utilization efficiency.

The ideal ammonium/nitrate ratio for plant growth varies based on the growth stage, genotype, environmental factors, and availability of inorganic nitrogen [10, 11]. Different ammonium/nitrate ratios strongly affect photosynthesis, growth and yield [12]. Despite the advantage of lower energy demand during ammonium assimilation, its phytotoxic effects become pronounced at high concentrations [13, 14]. Many studies have demonstrated that the combined application of nitrate and ammonium can effectively mitigate the toxic effects of ammonium [15–17]. Hence, it is necessary to consider the proportions of nitrate utilization and ammonium utilization. Given that the concentration of inorganic nitrogen could significantly affect the nitrogen utilization efficiency [18], quantification of the nitrogen accumulation derived from nitrate/ammonium utilization for plants grown under different ammonium/nitrate ratios can be used to assess the nitrogen utilization coefficients of ammonium and nitrate, thereby optimizing the supply of inorganic nitrogen during the growth stage.

The ^15^N labeling technique ensures a notable difference in ^15^N atom% between the ^15^N-labeled fertilizer and the background [19]. The ^15^N labeling method is recognized as the sole direct technique for assessing nitrogen uptake from labeled fertilizers [19–22]. In addition, this technique has been used to trace the fate of nitrogen within the plant–soil system [19, 23]. Hence, under hydroponic conditions, the amount of nitrogen accumulation derived from ammonium utilization can be quantified when the ^15^N-labeled ammonium is applied. As a result, the nitrogen accumulation amount derived from nitrate utilization can be determined indirectly when nitrate and ammonium are provided as the sole nitrogen sources in the nutrient solution.

Compared with nitrate or ammonium alone, the combined application of ammonium and nitrate can significantly improve the photosynthetic capacity [24–26]. Research indicates that the ammonium/nitrate ratio significantly affects plants’ net photosynthetic rate, likely due to the interaction between nitrogen metabolism and carbon assimilation [24, 27–29]. Excessive nitrate assimilation may cause competition for photosynthetic electron flow between carbon and nitrogen metabolism [30–32], thereby weakening the photosynthetic capacity of plants. In contrast, exposure to elevated ammonium concentrations has been shown to inhibit the O_2_-evolution complex [33] and impair photosystem II (PSII) efficiency [34, 35], ultimately leading to poor photosynthesis. Plant chlorophyll fluorescence is directly correlated with photosynthesis processes and can indicate the extent of photosynthetic damage due to abiotic stress [36–38]. Analyzing chlorophyll fluorescence parameters can elucidate the internal mechanisms by which varying ammonium/nitrate ratios influence photosynthesis.

*Coix lacryma-jobi *L*.* (*Coix*) is widely cultivated as a high-value economic crop and natural medicinal plant [39, 40]. In China, *Coix* is cultivated over an area of about 73,000 hectares, producing 220,000 tons of grain annually [41]. The southwest region of Guizhou Province is the leading producer and distribution hub for *Coix* in China [40, 41]. However, for *Coix*—an underutilized cereal with high nutritional and medicinal value—the interplay between nitrogen source and photosynthetic performance remains unknown, limiting the development of precision cultivation practices. In this study, *Coix* seedlings were exposed to varying ammonium/nitrate ratios, maintaining a total inorganic nitrogen concentration of 2 mM for each treatment. This study examined how varying ammonium/nitrate ratios affect the growth, photosynthesis, chlorophyll fluorescence, nitrogen and carbon content, and ^15^N values in *Coix* seedlings. The main aims of this study were (1) to elucidate the mechanisms underlying the synergistic effects between inorganic nitrogen utilization and carbon assimilation in *Coix* seedlings grown under different ammonium/nitrate ratios and (2) to quantify the nitrogen utilization efficiency of nitrate and ammonium over a larger time scale for *Coix* seedlings.

## Methods

### Plant materials and experimental treatments

Seeds of *Coix lacryma-jobi* L. ‘Yizhu 1’ were germinated in pre‑moistened sponge cubes (2 cm × 2 cm × 2 cm; one seed per cube) within trays in an artificial greenhouse. Seed-containing sponge cubes were misted four times daily to maintain moisture. The greenhouse conditions were as follows: a 12-h photoperiod with day/night temperatures of 26 ± 2 °C and 20 ± 2 °C, respectively; a photosynthetic photon flux density of 500 ± 20 µmol m^− 2^ s^− 1^; and a relative humidity of 55–60%.

After two weeks, vigorous seedlings were transplanted into plastic containers (43 cm × 19 cm × 14 cm), each equipped with a polystyrene foam board containing four holes (2 cm × 2 cm, spaced 10 cm apart). One seedling was inserted into each hole, with sponge material supporting the base. Containers were filled with 1/8-strength Hoagland nutrient solution [42] to 50% of their volume, such that the bottom of the foam board remained near the solution surface. The nutrient solution had a pH of 7.3 ± 0.1. The nutrient solution was continuously aerated using an air pump with a maximum output of 3.4 L min^− 1^ and was completely renewed every two days. After two weeks of growth, the nutrient solution was replaced with 1/4‑strength Hoagland solution (pH 7.3 ± 0.1) at the same volume, and the same aeration and renewal regime was maintained.

After an additional week, uniformly grown seedlings at the four‑leaf stage were subjected to hydroponic treatments with different ammonium/nitrate ratios: 10/90, 30/70, 50/50, and 70/30. Given that nitrate and ammonium typically coexist in natural soil environments, the artificially pure treatments (0/100 and 100/0 ammonium/nitrate ratios) were omitted in this study. The nutrient mixture contained 2 mM nitrogen (with different ammonium/nitrate ratios), 1 mM MgSO_4_·7H_2_O, 0.125 mM KH_2_PO_4_, 2.5 mM KCl, 2 mM CaCl_2_·2H_2_O, 0.1875 mM K_2_SO_4_, 50 µM FeEDTA, 25 µM H_3_BO_3_, 2 µM MnSO_4_·1H_2_O, 2 µM ZnSO_4_·7H_2_O, 0.1 µM CuSO_4_·5H_2_O, 0.04 µM CoCl_2_·6H_2_O, and 0.1 µM Na_2_MoO_4_·2H_2_O. In this study, ¹⁵N-labeled ammonium chloride (¹⁵NH₄Cl, 10 atom% ^15^N) was employed as the tracer source for ammonium, with a δ^15^N value of 29222.0‰. Unlabeled sodium nitrate (NaNO_3_) of natural ¹⁵N abundance served as the nitrate, exhibiting a δ^15^N value of 22.2‰. This experimental design enables clear distinction between nitrogen uptake pathways, as the elevated ^15^N signature observed in plant tissues is exclusively attributable to the labeled ammonium. Consequently, the nitrogen accumulation amount derived from ammonium utilization could be quantified directly, while the nitrogen accumulation amount derived from nitrate utilization was determined indirectly.

Each treatment consisted of three replicates (*n* = 3), with individual seedling serving as a replicate. Each seedling was grown individually in a plastic pot (16 cm × 13 cm × 8 cm) containing 1 L of the respective nutrient solution (pH 7.3 ± 0.1). Each plastic pot was covered with a polystyrene foam board having a single central hole (2 cm × 2 cm). The seedling was placed in the hole, and the basal part of the shoot was surrounded by sponge material. The solution was continuously aerated (air pump, maximum flow 3.4 L min^− 1^) and completely renewed every two days. The treatments lasted for two weeks.

At the start of the experiment, three seedlings at the four-leaf stage and with uniform growth status were selected to determine the dry weight, nitrogen content, and δ^15^N values of both shoots and roots. The average dry weight of shoots and roots for three seedlings was comparable to their initial dry weight as detailed in Additional file 1. The initial nitrogen contents of the shoots and roots of three seedlings were approximately equal to those at the experiment’s onset, as detailed in Additional file 1. The initial δ^15^N values of the shoots and roots of three seedlings were approximately equal to those at the experiment’s onset, as detailed in Additional file 1.

### Measurement of growth

After two weeks of culture, seedlings of *Coix lacryma-jobi* L. at the early vegetative stage were harvested; the term “*Coix* seedlings” hereafter refers specifically to plants at this developmental stage. Following dissection into shoots and roots, all samples were subjected to an initial 30-minute heat treatment at 105 °C, followed by drying at 70 °C until a constant weight was achieved. The dried samples were then ground into a fine powder.

### Determination of chlorophyll content and photosynthetic capacity

At the final harvest, the chlorophyll (Chl) content of the second fully expanded leaf of each *Coix* seedling was determined via a SPAD-502Plus chlorophyll meter (Konica Minolta, Tokyo, Japan). The photosynthetic capacity measurements of the second fully expanded leaf of each *Coix* seedling were performed with a LI-6800 photosynthesis system (LI-COR, Lincoln, USA). The gas flow rate, photosynthetically active radiation, CO_2_ concentration, relative humidity, and leaf temperature during the measurements were 500 µmol s^− 1^, 500 µmol m^− 2^ s^− 1^, 400 µmol m^− 2^ s^− 1^, 55% and 27.0 °C, respectively. The net photosynthetic rate (Pn), stomatal conductance (Gs), and transpiration rate (Tr) of the second fully expanded leaf of each *Coix* seedling were measured using a 6800–01 A fluorescence leaf chamber (2 cm^2^ aperture), ensuring the leaf fully covered the aperture. Measurements were taken between 09:00 and 11:00.

### Chlorophyll fluorescence measurements

Chlorophyll fluorescence measurements were conducted on the second fully expanded leaf of each *Coix* seedling using a LI-6800 photosynthesis system (LI-COR, Lincoln, USA). After 1 hour of dark adaptation, the initial fluorescence (F_o_) and maximum fluorescence (F_m_) of each *Coix* seedling were measured, allowing for the calculation of maximum quantum yield of photosystem II (F_v_/F_m_) using Eq. (1). While measuring the Pn of *Coix* seedlings, the maximum fluorescence (F_m_’), basic fluorescence post-induction (F_o_’), and steady-state fluorescence yield (F_s_) were simultaneously recorded in the light-adapted state. The effective quantum yield of photosystem II (Φ_PSII_), photochemical quenching coefficient (qP), nonphotochemical quenching coefficient (NPQ), and electron transport rate (ETR) were calculated using Eqs. (2, 3, 4 and 5):

1 $$\frac {\text F_\text v}{\text F_\text m}=\frac{({{\text F_\text m}}-{{\text F_\text o}})}{{{\text F_\text m}}}$$

2 $${\Phi _{{\mathrm{PSII}}}}=\frac{({{\mathrm{F}_\text m{'}}} - {{\mathrm{F}_\text s}})}{{\,{\text F_\text m{'}}}}$$

3 $${\mathrm{qP}}=\frac{({\text F_\text m{'}} - {\text F_\text s})}{({\text F_\text m{'}} - {\text F_\text o{'}})}$$

4 $${\mathrm{NPQ}}=\frac{({\mathrm{Fm}} - {\text F_\text m{'}})}{{\,\text F_ \text m{'}}}$$

5 $${\mathrm{ETR}}={\mathrm{PPFD}} \times {\Phi _{{\mathrm{PSII}}}} \times 0.85 \times {\mathrm{0}}{\mathrm{.5}}$$

### Analysis of elements and determination of δ^15^N

The total nitrogen and carbon contents of the dried shoots and roots were measured via an elemental analyzer (vario MACRO cube, Germany). The δ^15^N values for both shoots and roots were measured using a MAT-253 gas isotope ratio mass spectrometer from Germany. The δ^15^N values were calculated using Eq. (6):

6 $$\delta^{15}\mathrm{N} (‰) = \left( \frac{R_{\mathrm{sample}}}{R_{\mathrm{standard}}} - 1 \right) \times 1000$$

where *R*_sample_ denotes the nitrogen isotope ratio of the plant material and *R*_standard_ denotes the isotope ratio of a known standard (N_2_ in air). For the determination of initial δ^15^N values in shoots and roots of the *Coix* seedling, the internal standard was the IAEA N_2_ (δ^15^*N* = 20.3‰) of the International Atomic Energy Agency. During subsequent analysis of the δ^15^N values in the shoots and roots of the *Coix* seedlings at the harvest stage, calibration was performed via IAEA 311 (2.05 atom% ^15^N) from the same institution.

### ^15^N analysis of shoots and roots

After the δ^15^N values of the shoots and roots were determined, the ^15^N atom percentages in the shoots and roots were calculated according to Eq. (7):

7 $${}^\mathrm{15}\mathrm N_{\mathrm{shoot}\;\mathrm{or}\;\mathrm{root}}\;\mathrm{atom}\%\mathrm{=}\left(\frac{R_\mathrm{sample}}{(R_\mathrm{sample}+1)}\right)\times100$$

The nitrogen contributions in shoots and roots from ^15^N-labeled fertilizer were calculated using Eqs. (8, 9):

8 $${\mathrm N}_{\mathrm{shoot}}\mathrm{dff(}\%)\mathrm{=}\frac{{}^\mathrm{15}\mathrm N_\mathrm{shoot}-0.3663\%}{{}^\mathrm{15}\mathrm N_\mathrm{fertilizer}-0.3663\%}\times100$$

9 $${\mathrm N}_{\mathrm{root}}\mathrm{dff(}\%)\mathrm{=}\frac{{}^\mathrm{15}\mathrm N_\mathrm{root}-0.3663\%}{{}^\mathrm{15}\mathrm N_\mathrm{fertilizer}-0.3663\%}\times100$$

where N_shoot_dff and N_root_dff denote the nitrogen in shoots and roots, respectively, sourced from ^15^N-labeled fertilizer. The ^15^N abundance in labeled fertilizer was 10%, and 0.3663% refers to the natural ^15^N abundance. The amounts of nitrogen in shoots and roots from ^15^N-labeled fertilizer were calculated using Eqs. (10, 11):

10 $$\mathrm{AN}_\mathrm{shoot}=\frac{\left(\mathrm{N}_\mathrm{shoot}\mathrm{dff} \times\mathrm{DW}_\mathrm{shoot} \times \mathrm{N}_\mathrm{shoot}\right)}{\mathrm{M}_\mathrm{a}}$$

11 $$\mathrm{AN}_\mathrm{root}=\frac{\left(\mathrm{N}_\mathrm{root}\mathrm{dff} \times\mathrm{DW}_\mathrm{root} \times \mathrm{N}_\mathrm{root}\right)}{\mathrm{M}_\mathrm{a}}$$

where AN_shoot_ and AN_root_ denote the amount of nitrogen in shoots and roots, respectively, sourced from ^15^N-labeled fertilizer. The molar mass of ammonium nitrogen (M_a_) is 14.1 g/mol. N_shoot_ and N_root_ represent the nitrogen contents in shoots and roots, respectively.

The nitrogen accumulation amount derived from ammonium utilization in whole *Coix* seedlings (NAA_ammonium_) was calculated using Eq. (12):

12 $${\mathrm{NAA}}_{\mathrm{ammonium}}\mathrm{=}\mathrm A{\mathrm N}_\mathrm{shoot}+{\mathrm{AN}}_{\mathrm{root}}$$

### Total nitrogen and total carbon accumulations of the whole *Coix* seedlings

After the nitrogen content and carbon contents in shoots and roots of *Coix* seedlings at the harvest stage were determined, the total nitrogen and total carbon accumulations of the whole *Coix* seedlings were calculated using Eqs. (13, 14):

13 $$\mathrm{TN} = \frac{ \mathrm{DW_{shoot}} \times \mathrm{N_{shoot}} \times (1 - \mathrm{N_{shoot}dff}) + \mathrm{DW_{root}} \times \mathrm{N_{root}} \times (1 - \mathrm{N_{root}dff}) }{ \mathrm{M} } + \mathrm{NAA_{ammonium}}$$

14 $$\mathrm T\text{C =}\frac{({\mathrm{DW}}_{\mathrm{shoot}}\times{\mathrm{C}}_{\mathrm{shoot}}{\mathrm{+DW}}_{\mathrm{root}}\times{\mathrm{C}}_{\mathrm{root}}\mathrm{)}}{{\mathrm M}_{\mathrm c}}$$

where TN and TC are the total nitrogen and total carbon of the whole *Coix* seedlings, respectively. C_shoot_ and C_root_ represent the carbon contents in shoots and roots, respectively. M, with a molar mass of 14 g/mol, represents naturally abundant nitrogen. M_c_, with a molar mass of 12 g/mol, represents naturally abundant carbon. 

### Nitrogen accumulation derived from nitrate utilization in the whole *Coix* seedlings

At harvest, the total nitrogen in *Coix* seedlings comprised the initial nitrogen accumulation, nitrogen from ammonium utilization, and nitrogen from nitrate utilization. Hence, on the basis of the law of mass conservation, the nitrogen accumulation amount derived from nitrate utilization in the whole *Coix* seedlings (NAA_nitrate_) was calculated using Eq. (15):

15 $${\mathrm{NAA}}_{\mathrm{nitrate}} = \mathrm{TN} - {\mathrm{NAA}}_{\mathrm{ammonium}} - \frac{ {\mathrm{DW}}_{\mathrm{shoot}0} \times {\mathrm{N}}_{\mathrm{shoot}0} + {\mathrm{DW}}_{\mathrm{root}0} \times {\mathrm{N}}_{\mathrm{root}0} }{ \mathrm{M} }$$

where DW_shoot0_ and DW_root0_ represent the initial dry weights of shoots and roots, respectively, and N_shoot0_ and N_root0_ denote their initial nitrogen contents. The standard error (SE) of NAA_nitrate_ was calculated via the error propagation formula [43].

### Proportion of ammonium and nitrate utilization

After NAA_ammonium_ and NAA_nitrate_ were determined, the proportion of ammonium utilization (*f*_A_) by the *Coix* seedlings during the whole culture period was calculated using Eq. (16):

16 $$f_\text A=\frac{\mathrm{NAA}_{\mathrm{ammonium}}}{({\mathrm{NAA}}_{\mathrm{ammonium}}\mathrm{+}\mathrm N\mathrm A{\mathrm A}_{\mathrm{nitrate}}\mathrm{)}}$$

Because the amount of nitrogen accumulation of whole *Coix* seedlings during the whole culture period was derived from ammonium and nitrate utilization, the proportion of nitrate utilization (*f*_N_) by the *Coix* seedlings during the whole culture period was calculated using Eq. (17):

17 $$f_{\text N}=1-f_{\mathrm A}$$

The standard errors (SEs) of *f*_A_ and *f*_N_ were calculated via the error propagation formula [43].

### Nitrogen utilization coefficients of ammonium and nitrate

The nitrogen utilization coefficient of ammonium (NUC_ammonium_) of the *Coix* seedlings was the ratio of NAA_ammonium_ relative to the total ammonium supply during the whole culture period. The nitrogen utilization coefficient of nitrate (NUC_nitrate_) of the *Coix* seedlings was the ratio of NAA_nitrate_ relative to the total nitrate supply during the whole culture period. NUC_ammonium_ and NUC_nitrate_ were calculated using Eqs. (18, 19):

18 $${\mathrm{NUC}}_{\mathrm{ammonium}}\mathrm{=}\frac{{\mathrm{NAA}}_{\mathrm{ammonium}}}{{\mathrm{(}}_{\mathrm{Cammonium}}\times\mathrm{V}\times\mathrm{n)}}$$

19 $${\mathrm{NUC}}_{\mathrm{nitrate}}\mathrm{=}\frac{{\mathrm{NAA}}_{\mathrm{nitrate}}}{(\mathrm{c}_{\mathrm{nitrate}}\times\mathrm{V}\times\mathrm{n})}$$

where c_ammonium_ and c_nitrate_ represent the concentrations of ammonium and nitrate in the culture solution, respectively. V represents the volume of the culture solution, and n represents the number of times the culture solution was replaced during the whole culture period. The standard error (SE) of NUC_nitrate_ was calculated via the error propagation formula [43].

### Statistical analysis

All results are expressed as the mean ± standard deviation (SE) of three replicates. Significance testing was conducted using one-way analysis of variance (ANOVA) and Duncan’s test (*p* < 0.05). Data Processing System (DPS) software (version 9.01, Hangzhou Ruifeng Information Technology Co., Ltd., Hangzhou, China) was used for all analyses.

## Results

### Growth parameters

The growth of *Coix* seedlings was significantly influenced by the ammonium/nitrate ratio (Table 1). The variation in the ammonium/nitrate ratio induced an approximately 10% shift in the biomass of *Coix* seedlings. As the ammonium/nitrate ratio increased, the dry weight of the roots of *Coix* seedlings initially increased significantly but subsequently decreased significantly. However, the ammonium/nitrate ratio did not significantly affect the dry weight of the shoots of *Coix* seedlings. An optimal ammonium/nitrate ratio is essential for significantly enhancing the growth of *Coix* seedling.

**Table 1:** The biomass of *Coix lacryma-jobi* L. seedlings grown under different ammonium/nitrate ratios

Parameters	NH_4_^+^:NO_3_^−^ ratios				
	10:90	30:70		50:50	70:30
Dry weight (g/plant)	4.538 ± 0.060^bc^	5.095 ± 0.130^ab^		5.168 ± 0.280^a^	4.492 ± 0.140^c^
Shoot DW (g)	3.398 ± 0.085^a^	3.816 ± 0.095^a^		3.788 ± 0.219^a^	3.371 ± 0.133^a^
Root DW (g)	1.140 ± 0.028^bc^	1.279 ± 0.049^ab^		1.380 ± 0.061^a^	1.121 ± 0.021^c^

Each value is expressed as the mean ± SE (*n* = 3)

Means with different letters in each row differ significantly according to Duncan’s test (*p* < 0.05)

### Photosynthesis, SPAD, and Chl fluorescence

The ammonium/nitrate ratio significantly affected the gas exchange parameters of *Coix* seedlings. As shown in Table 2, the ammonium/nitrate ratios of 30/70 and 50/50 significantly improved the stomatal conductance, transpiration rate and net photosynthetic rate of *Coix* seedlings. However, the lowest and highest ammonium/nitrate ratios (10/90 and 70/30) had negative effects on the Gs, Tr and Pn of *Coix* seedlings. Interestingly, increasing the ammonium/nitrate ratio significantly increased the SPAD values of *Coix* seedlings. Interestingly, although increasing the ammonium/nitrate ratio significantly elevated the SPAD values in *Coix* seedlings, it did not translate into enhanced photosynthetic performance. Specifically, despite no significant difference in chlorophyll content between the ammonium/nitrate ratios of 50/50 and 70/30, the net photosynthetic rate decreased by approximately 10% at the 70/30 ratio.

**Table 2:** The photosynthetic parameters, chlorophyll fluorescence, and SPAD of *Coix lacryma-jobi* L. seedlings grown under different ammonium/nitrate ratios

Parameters	NH_4_^+^:NO_3_^−^ ratios			
	10:90	30:70	50:50	70:30
Pn (µmol m^− 2^ s^− 1^)	18.865 ± 0.217^b^	21.544 ± 0.406^a^	20.798 ± 0.316^a^	18.541 ± 0.434^b^
Gs (mol m^− 2^ s^− 1^)	0.132 ± 0.005^b^	0.152 ± 0.006^a^	0.155 ± 0.002^a^	0.133 ± 0.002^b^
Tr (mmol m^− 2^ s^− 1^)	2.208 ± 0.082^b^	2.542 ± 0.078^a^	2.588 ± 0.040^a^	2.232 ± 0.027b
Fv/Fm	0.798 ± 0.003^a^	0.790 ± 0.003^a^	0.791 ± 0.002^a^	0.792 ± 0.001^a^
Φp	0.480 ± 0.003^b^	0.519 ± 0.011^a^	0.506 ± 0.009^a^	0.471 ± 0.006^b^
qP	0.797 ± 0.012^a^	0.803 ± 0.012^a^	0.802 ± 0.020^a^	0.773 ± 0.005^a^
NPQ	0.893 ± 0.031^a^	0.621 ± 0.040^b^	0.682 ± 0.018^b^	0.882 ± 0.041^a^
ETR	101.189 ± 0.691^b^	109.452 ± 2.184^a^	106.555 ± 1.836^a^	99.237 ± 1.193^b^
SPAD	40.733 ± 1.312^c^	45.700 ± 0.416^b^	46.700 ± 0.751^ab^	48.433 ± 0.145^a^

Each value is expressed as the mean ± SE (*n* = 3)

Means with different letters in each row differ significantly according to Duncan’s test (*p* < 0.05)

Chlorophyll fluorescence analysis helped elucidate the internal mechanisms by which varying ammonium/nitrate ratios influence photosynthesis in *Coix* seedlings. The *Coix* seedlings grown at the 30/70 and 50/50 ratios of ammonium/nitrate had significantly greater Φp and ETR and significantly lower NPQ values. Our results showed that the ammonium/nitrate ratio exerted a significant influence on the photochemical activity of PSII.

### Nitrogen content and carbon content in shoots and roots

**Fig. 1:**
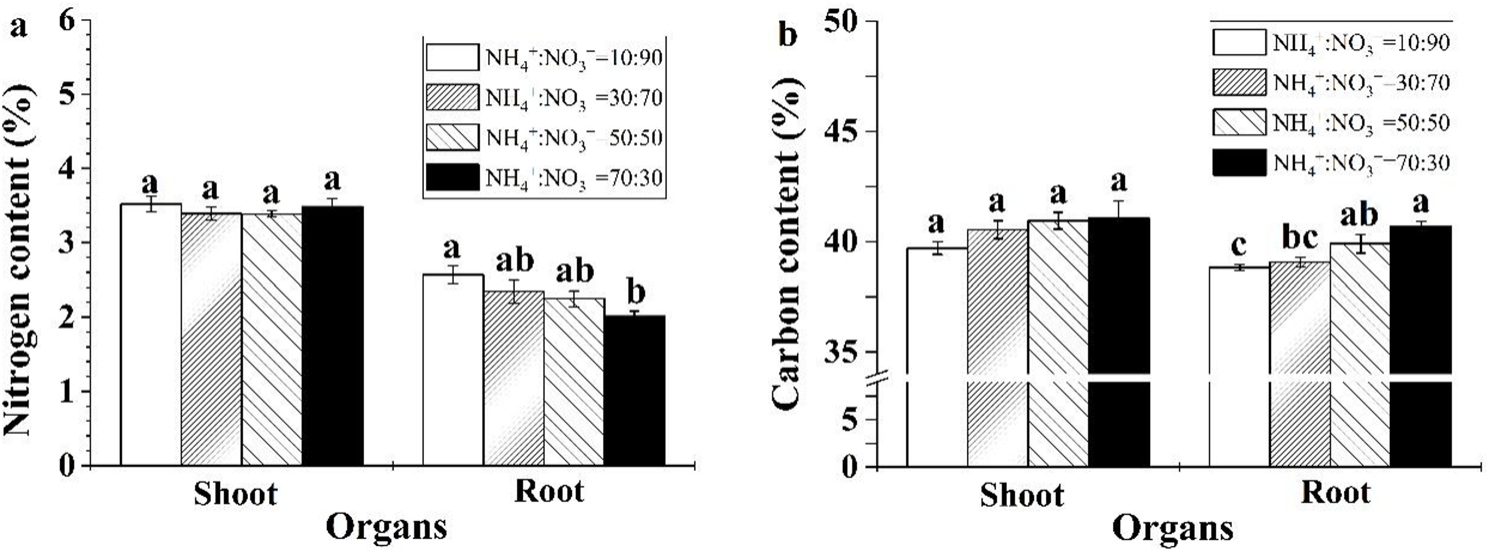
Nitrogen content (**a**) and carbon content (**b**) of *Coix lacryma-jobi* L. seedlings grown under different ammonium/nitrate ratios. Note: Values are presented as means ± SEs (*n* = 3). Different small letters indicate significant differences according to Duncan’s test (*P* < 0.05)

The nitrogen content in the shoots and roots of *Coix* seedlings responded differently to different ammonium/nitrate ratios (Fig. 1a). An increase in the ammonium/nitrate ratio did not significantly increase the nitrogen content of the shoots of the *Coix* seedlings. Interestingly, the nitrogen content of the roots decreased significantly with increases in the ammonium/nitrate ratio. Relative to the 10/90 ammonium/nitrate ratio, a reduction of approximately 20% in nitrogen content of roots was observed in *Coix* seedlings grown at the 70/30 ratio. The ammonium/nitrate ratio did not significantly affect the carbon content of the shoots of the *Coix* seedlings. However, with increases in the ammonium/nitrate ratio, the carbon content of roots significantly increased (Fig. 1b).

### Total nitrogen and total carbon of the whole *Coix* seedlings

**Fig. 2:**
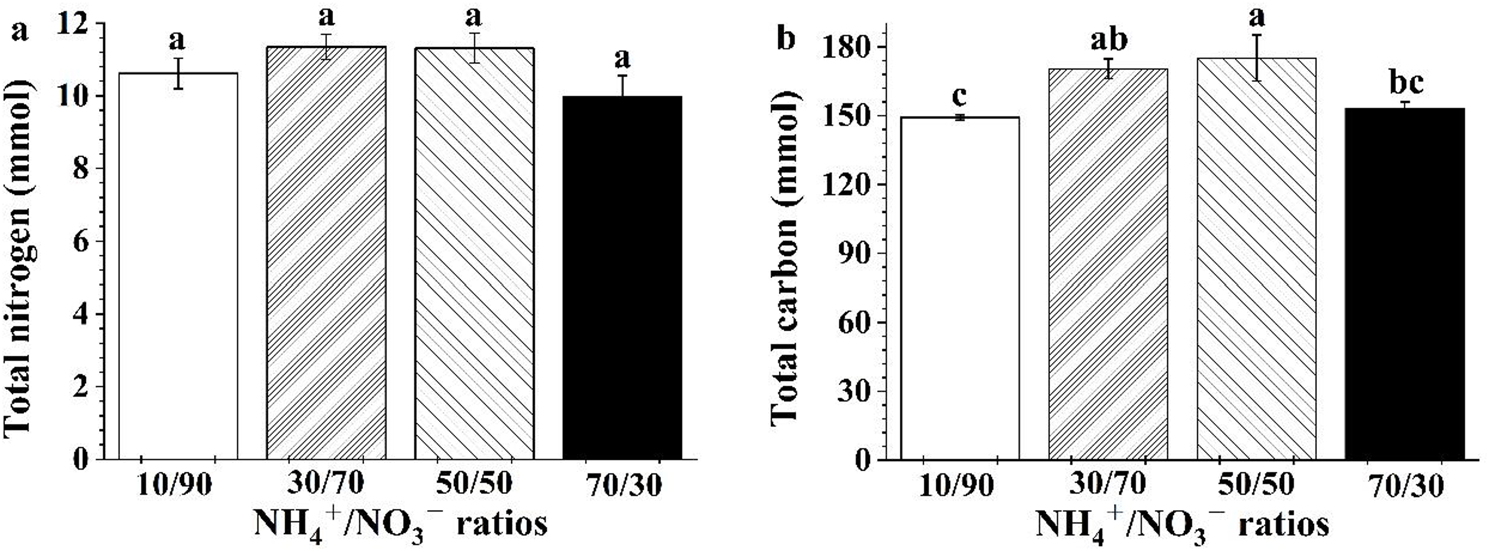
Total nitrogen (**a**) and total carbon accumulations (**b**) of whole *Coix lacryma-jobi* L. seedlings grown under different ammonium/nitrate ratios. Note: Values are presented as means ± SEs (*n* = 3). Different small letters indicate significant differences according to Duncan’s test (*P* < 0.05)

After two weeks of cultivation, the total nitrogen accumulation in *Coix* seedlings showed no significant variation across the four ammonium/nitrate ratios. The *Coix* seedlings exhibited the lowest total nitrogen accumulation at an ammonium/nitrate ratio of 70/30 (Fig. 2a). The total carbon accumulation in whole *Coix* seedlings was significantly influenced by the ammonium/nitrate ratio. With increases in the ammonium/nitrate ratio, the total carbon accumulation in whole *Coix* seedlings initially significantly increased but then markedly decreased (Fig. 2b). Changes in the ammonium/nitrate ratio caused a shift in carbon accumulation in excess of 10% for *Coix* seedlings.

### Isotopic nitrogen composition of shoots and roots

**Fig. 3:**
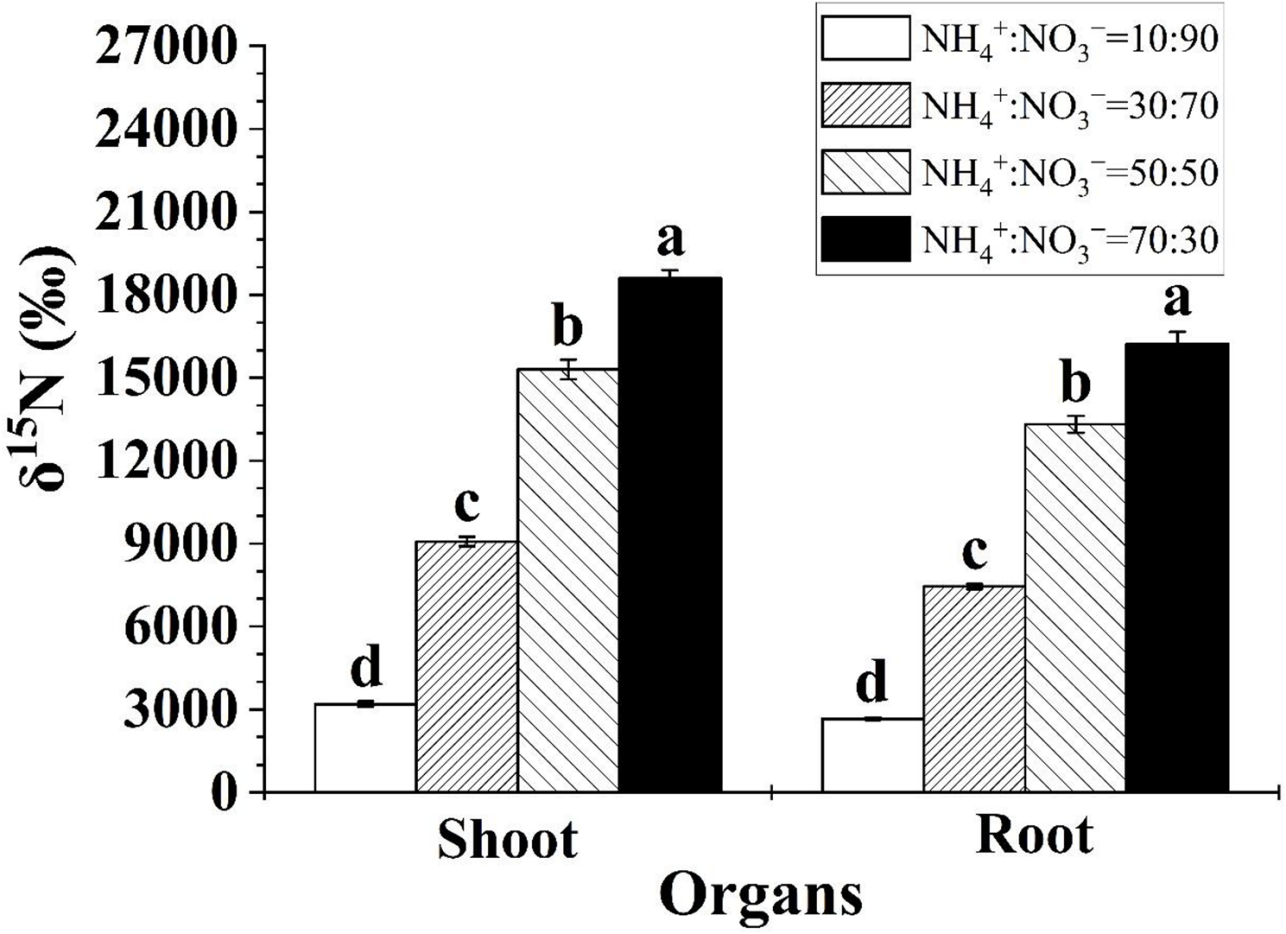
δ^15^N in the shoots and roots of *Coix lacryma-jobi* L. seedlings grown under different ammonium/nitrate ratios. Note: Values are presented as means ± SEs (*n* = 3). Different small letters indicate significant differences according to Duncan’s test (*P* < 0.05)

There were differences in the organ-level δ^15^N values of the *Coix* seedlings grown under different ammonium/nitrate ratios (Fig. 3). In general, the shoots consistently presented greater ^15^N enrichment than did the roots among the four ammonium/nitrate ratios. In addition, the δ^15^N values of both the shoots and roots significantly increased as the ammonium/nitrate ratio increased. Elevated ammonium availability contributed to enriching ^15^N in the *Coix* seedlings.

### Nitrogen accumulation derived from ammonium and nitrate utilization

**Fig. 4:**
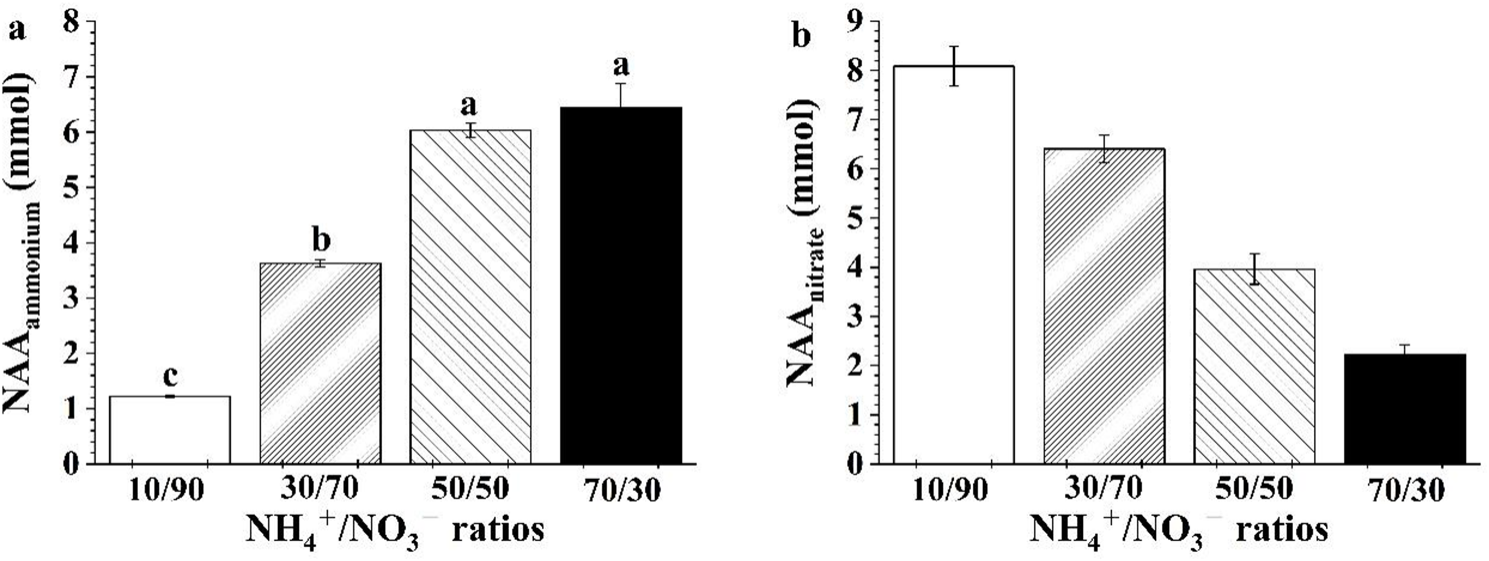
Nitrogen accumulation derived from ammonium utilization (**a**) and nitrate utilization (**b**) in whole *Coix lacryma-jobi* L. seedlings grown under different ammonium/nitrate ratios. Note: NAA_ammonium_, defined as the nitrogen accumulation amount derived from ammonium utilization. NAA_nitrate_, defined as the nitrogen accumulation amount derived from nitrate utilization. Values are presented as means ± SEs (*n* = 3). Different small letters indicate significant differences according to Duncan’s test (*P* < 0.05). The error bars of NAA_nitrate_ were calculated by the error propagation formula

The NAA_ammonium_ of whole *Coix* seedlings was notably influenced by the ammonium/nitrate ratio (Fig. 4a). The NAA_ammonium_ of whole *Coix* seedlings increased linearly as the ammonium proportion in the mixed nitrogen source ranged from 10% to 70%. No notable difference in the NAA_ammonium_ was found in whole *Coix* seedlings at ammonium/nitrate ratios of 50/50 and 70/30. As the ammonium/nitrate ratio increased, the NAA_nitrate_ in whole *Coix* seedlings markedly decreased (Fig. 4b). The NAA_nitrate_ in whole *Coix* seedlings was influenced by the nitrate concentration in the solution. The NAA_nitrate_ in whole *Coix* seedlings under the 70/30 ammonium/nitrate ratio was less than 30% of that under the 10/90 ratio.

### Proportions of ammonium and nitrate utilization

**Fig. 5:**
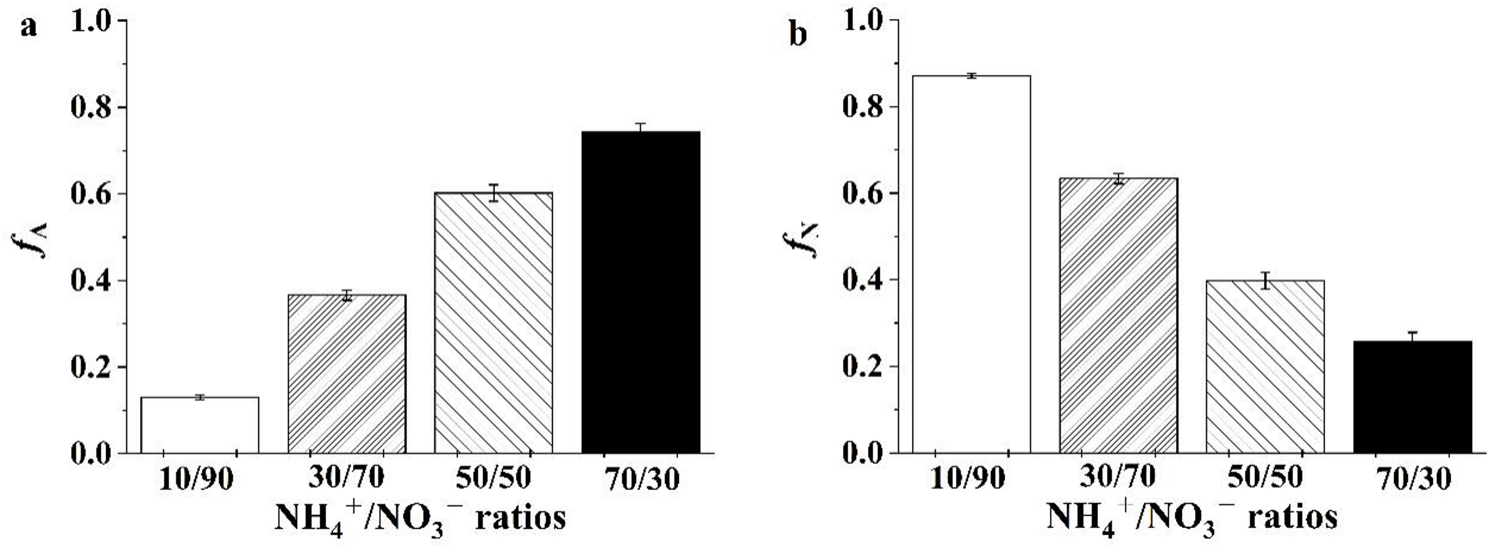
Proportions of ammonium utilization (**a**) and nitrate utilization (**b**) by the *Coix lacryma-jobi* L. seedlings grown under different ammonium/nitrate ratios. Note: *f*_A_, defined as the proportion of ammonium utilization. *f*_N_, defined as the proportion of nitrate utilization. The error bars were calculated by the error propagation formula

Increasing the ammonium/nitrate ratio increased the proportion of ammonium utilized by the *Coix* seedlings (Fig. 5a). When the ammonium/nitrate ratio reached 70/30, over 70% of the nitrogen in *Coix* seedlings was derived from ammonium utilization. In contrast, at the ammonium/nitrate ratio of 10/90, nearly 90% of the nitrogen in *Coix* seedlings was derived from nitrate utilization (Fig. 5b). Generally, the proportion of ammonium/nitrate utilization by the *Coix* seedlings mainly depended on their availability in the solution.

### Nitrogen utilization coefficients of ammonium and nitrate

**Fig. 6:**
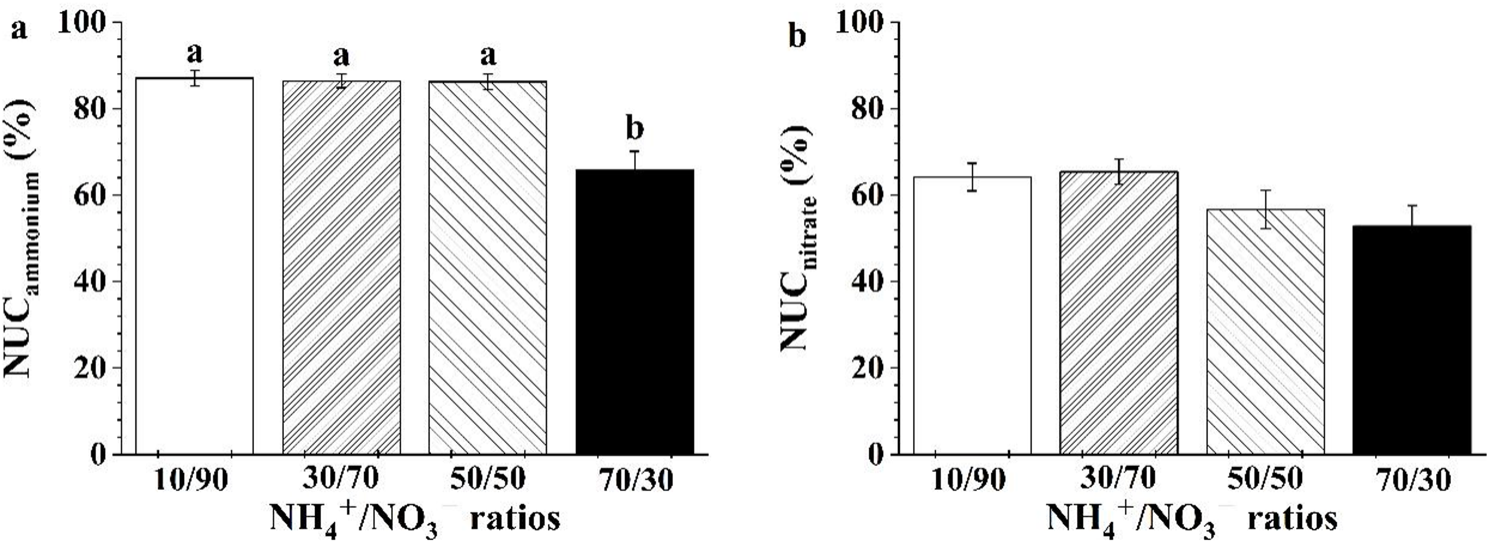
The utilization coefficients of ammonium (**a**) and nitrate (**b**) in *Coix lacryma-jobi* L. seedlings grown under different ammonium/nitrate ratios. Note: NUC_ammonium_, defined as the nitrogen utilization coefficient of ammonium. NUC_nitrate_, defined as the nitrogen utilization coefficient of nitrate. Values are presented as means ± SEs (*n* = 3). Different small letters indicate significant differences according to Duncan’s test (*P* < 0.05). The error bars of NUC_nitrate_ were calculated by the error propagation formula

The NUC_ammonium_ of *Coix* seedlings only significantly decreased at the ammonium/nitrate ratio of 70/30 (Fig. 6a). Compared with the other ammonium/nitrate ratios, the highest ammonium/nitrate ratio resulted in a reduction of approximately 20% in the NUC_ammonium_ of *Coix* seedlings. Varying the ammonium/nitrate ratio between 10/90 and 50/50 did not significantly impact the NUC_ammonium_ of *Coix* seedlings. The NUC_nitrate_ of *Coix* seedlings slightly decreased when the ammonium/nitrate ratio reached 50/50 (Fig. 6b). Generally, both the NUC_ammonium_ and NUC_nitrate_ of *Coix* seedlings reached their minimum values at the ammonium/nitrate ratio of 70/30.

## Discussion

The main forms of inorganic nitrogen utilized by plants are nitrate and ammonium. The growth of plants is significantly influenced by the levels of inorganic nitrogen and the ammonium/nitrate ratio. Given that aerobic agricultural soil typically contains around 5 mM of inorganic nitrogen [1], reducing the concentration of inorganic nitrogen and optimizing the ammonium/nitrate ratio could contribute to improving the nitrogen utilization efficiency of *Coix* seedlings. Optimizing the ammonium/nitrate ratio significantly influenced the growth of *Coix* seedlings when the total nitrogen concentration was 2 mM (Table 1). Our study found that a 50/50 ammonium/nitrate ratio yielded the highest biomass in *Coix* seedlings. The biomass of *Coix* seedlings at the lowest and highest ammonium/nitrate ratios (10/90 and 70/30) was significantly lower than that at the 50/50 ratio. In general, a high ammonium concentration can significantly hinder the plant growth even if the nitrate is present in the nutrient solution [13, 44]. Additionally, excessive nitrate assimilation is not beneficial for the biomass accumulation because nitrate assimilation consumes more energy than ammonium assimilation [8–9]. Hence, a suitable ammonium/nitrate ratio is essential for significantly enhancing the growth of *Coix* seedlings.

Previous studies have suggested that the ammonium/nitrate ratio significantly influences photosynthesis in leaves [24, 27, 28]. This study yielded comparable findings (Table 2). An appropriate ammonium/nitrate ratio (30/70 or 50/50) significantly enhanced the photosynthetic capacity of *Coix* seedlings. The *Coix* seedlings presented the lowest net photosynthetic rate at an ammonium/nitrate ratio of 70/30, indicating that ammonium-induced stress was likely triggered at this ammonium concentration. Plants subjected to ammonium-induced stress usually exhibit significant stomatal restriction [45]. In addition, high concentrations of ammonium can inhibit the O_2_-evolution complex [33] and impair PSII efficiency [34, 35]. In this study, high concentrations of ammonium significantly decreased the Φp and ETR of *Coix* seedlings. Moreover, the NPQ of *Coix* seedlings was significantly greater at the highest ammonium/nitrate ratio than at the appropriate ammonium/nitrate ratios (30/70 and 50/50). Hence, high ammonium levels (70%) in a mixed nitrogen source are not beneficial for improving the photosynthesis of *Coix* seedlings. Moreover, high nitrate levels (90%) in a mixed nitrogen source are also not conducive to enhancing the photosynthetic capacity of *Coix* seedlings. Approximately 10% of the electron flux in illuminated leaves is allocated to nitrate reduction [46]. Hence, there might be competition for photosynthetic electron flow between carbon and nitrogen metabolism when nitrate is dominant in a mixed nitrogen source [30–32]. As a result, the reduction in ETR might be the cause of the decline in the net photosynthetic rate for *Coix* seedlings grown at the lowest ammonium/nitrate ratio [29, 47]. As shown in Fig. 1a, no significant difference was observed for the shoot nitrogen content of *Coix* seedlings grown under all ammonium/nitrate ratios. This suggests that the *Coix* seedlings grown at the lowest ammonium/nitrate ratio may prioritize nitrogen allocation for nitrogen metabolism enzymes (such as nitrate reductase and nitrite reductase) over chlorophyll synthesis, leading to reduced chlorophyll content (Table 2). The significantly lower chlorophyll content in *Coix* seedlings under the lowest ammonium/nitrate ratio led to severe excess light energy pressure on the photosynthetic units. This, in turn, triggered a strong photoprotective mechanism, thereby resulting in a significant increase in NPQ. Generally, the new carbon input of plants comes from the photosynthesis in leaves. The amount of total carbon accumulation in *Coix* seedlings could indicate their photosynthetic capacity. As shown in Fig. 2b, the lowest amount of total carbon accumulation in *Coix* seedlings was observed when nitrate made up 90% of the nitrogen source. The poor photosynthetic capacity of *Coix* seedlings at the lowest ammonium/nitrate ratio might be attributed to excessive nitrate assimilation because no significant difference was detected in the total nitrogen accumulation of *Coix* seedlings among all ammonium/nitrate ratios [8, 9].

On the basis of the ^15^N labeling technique, the uptake of the labeled ammonium nitrogen can be quantitatively assessed in *Coix* seedlings grown under varying ammonium/nitrate ratios [19–22]. With increasing ammonium concentration, the significant increase in the δ^15^N values in the shoots and roots of *Coix* seedlings could indicate an obvious increase in ammonium utilization. After the δ^15^N values of the shoots and roots of *Coix* seedlings are determined, the amount of nitrogen accumulation in whole *Coix* seedlings derived from ammonium utilization can be quantified via Eqs. (7)–(12). Raising the ammonium/nitrate ratio from 10/90 to 50/50 notably increased the NAA_ammonium_ of the whole *Coix* seedlings (Fig. 4a). However, the NAA_ammonium_ of the whole *Coix* seedlings showed no significant variation across ammonium/nitrate ratios ranging from 50/50 to 70/30. Hence, when ammonium supply surpassed a certain level, a greater supply of ammonium was not able to significantly increase the NAA_ammonium_ of the whole *Coix* seedlings. The 1.4 mM ammonium supply might exceed the demand of *Coix* seedlings for ammonium, suggesting a waste of ammonium [48].

In this study, the nitrogen accumulation in whole *Coix* seedlings was attributed to the utilization of nitrate and ammonium. Consequently, the nitrogen accumulation amount in whole *Coix* seedlings derived from nitrate utilization can be obtained indirectly by determining the NAA_ammonium_ and the nitrogen accumulation of the whole *Coix* seedlings at the beginning stage and harvest stage. After quantifying the NAA_ammonium_ and NAA_nitrate_ of whole *Coix* seedlings, the contribution of nitrate/ammonium utilization to the nitrogen accumulation of whole *Coix* seedlings grown under different ammonium/nitrate ratios can be evaluated. As shown in Fig. 5, the nitrogen accumulation of the whole *Coix* seedlings was due mainly to the utilization of the dominant inorganic nitrogen in the mixed nitrogen source, which indicated a strong flexibility in the uptake of inorganic nitrogen by *Coix* seedlings [3–5]. In general, the uptake of ammonium results in rhizosphere acidification, while the uptake of nitrate leads to rhizosphere alkalinization [49]. The difference in the proportion of ammonium utilization and nitrate utilization could affect the rhizosphere pH for *Coix* seedlings grown under different ammonium/nitrate ratios. However, the nutrient solution was completely renewed every 2 days in this study, which contributed to minimizing the short-term pH fluctuations. The proportion of nitrate utilization was markedly lower than that of ammonium under a 50/50 ratio of ammonium/nitrate, indicating a clear uptake preference for ammonium in *Coix* seedlings [8, 24]. Although *Coix* seedlings appears to prefer ammonium in terms of uptake quantity, the balanced supply with nitrate (50/50) represents its metabolic preference based on growth and carbon accumulation performance. Moreover, the maintenance of an equimolar ratio between nitrate and ammonium in agricultural soils serves to effectively minimize fluctuations in rhizosphere pH.

Efficient coordination between carbon assimilation and nitrogen metabolism has been demonstrated to be essential for maximizing plant growth [50]. At the balanced 50/50 ammonium/nitrate ratio, carbon accumulation in *Coix* seedlings was maximized. This can be explained by the synergistic effect of minimized energy expenditure for nitrogen assimilation (approximately 60% ammonium utilization) and a concomitant boost in photosynthetic capacity. High ammonium levels (70%) in the solution can trigger futile ammonium cycling, which is a highly energy-intensive process [51, 52]. A greater allocation of photosynthate to the roots may occur to sustain their growth [53, 54]. As a result, a significant increase in root carbon content was observed for *Coix* seedlings grown under the 70/30 ratio of ammonium/nitrate (Fig. 1b). Under the highest ammonium/nitrate ratio, over 70% of nitrogen in *Coix* seedlings was derived from ammonium assimilation. Although the elevated proportion of ammonium utilization significantly promotes the chlorophyll biosynthesis of *Coix* seedlings [55, 56], the substantial assimilation of ammonium may induce cytoplasmic acidification [57], which can inhibit the activity of key Calvin cycle enzymes such as Rubisco [58]. This restriction in downstream carbon fixation may reduce the energy utilization efficiency of the light reactions—evidenced by the significant declines in ETR and ΦPSII—and triggers a strong photoprotective mechanism, as indicated by a significant elevated NPQ. Ultimately, these responses lead to a significant decrease in the net photosynthetic rate of *Coix* seedlings. This physiological dysregulation of carbon–nitrogen balance may be a major factor contributing to the lower nitrogen use efficiency observed in this treatment group. As shown in Fig. 6, the NUC_ammonium_ of *Coix* seedlings significantly decreased when ammonium constituted 70% of the mixed nitrogen source. Moreover, the NUC_nitrate_ of *Coix* seedlings achieved the minimum value at the 70/30 ratio of ammonium/nitrate. Hence, the highest ammonium/nitrate ratio was not conducive to increasing the nitrogen utilization coefficient of both nitrate and ammonium in *Coix* seedlings. Among all the ammonium/nitrate ratios, NUC_ammonium_ was much greater than NUC_nitrate_ (Fig. 6). Although the NUC_nitrate_ of *Coix* seedlings slightly decreased when ammonium made up 50% of the nitrogen source, the NUC_ammonium_ of *Coix* seedlings did not significantly decrease with increasing ammonium concentration. Additionally, the total nitrogen accumulation of the whole *Coix* seedlings was almost the same when the ammonium/nitrate ratio was 30/70 and 50/50 (Fig. 2), indicating that increasing the ammonium concentration did not significantly reduce the nitrogen utilization coefficient. Furthermore, given that the rainfall events are very frequent during the growth stage of *Coix* seedlings and that the nitrate is prone to be lost from soils when it rains [6, 7], reducing the supply of nitrate will contribute to avoiding the potential loss of nitrate. An ammonium/nitrate ratio of 50/50 can be considered the optimal ammonium/nitrate ratio for improving the nitrogen utilization efficiency of *Coix* seedlings. Hence, quantifying the nitrogen accumulation derived from ammonium and nitrate utilization not only contributes to understanding the inorganic nitrogen utilization strategy in *Coix* seedlings grown under different ammonium/nitrate ratios but also offers a method to optimize inorganic nitrogen supply.

## Conclusions

Based on the ^15^N isotope tracer technique, the nitrogen accumulation amount derived from labeled ammonium nitrogen was directly quantified in *Coix* seedlings grown under different ammonium/nitrate ratios. Consequently, the nitrogen accumulation amount derived from nitrate utilization under the same conditions was calculated by difference. Our results indicated that an ammonium/nitrate ratio of 50/50 was optimal for *Coix* seedlings, yielding maximum carbon accumulation along with high ammonium use efficiency. While this finding is derived from a controlled seedling-stage study, and thus require further validation under field conditions and across the full growth cycle, it establishes a critical foundation. This integrated approach elucidates the synergy between nitrogen and carbon metabolism and provides a framework for precision nitrogen management, contributing to the advancement of sustainable cultivation practices for *Coix*. 

## Supplementary Information

Additional file 1.

Additional file 2.

## Data Availability

The data supporting the findings of this study are available from the corresponding author on reasonable request.
