# Supplementary material for: Elucidation of inorganic nitrogen utilization characteristics in Coix lacryma-jobi L. seedlings under variable ammonium/nitrate ratios via the 15N tracer technique
Source: BMC Plant Biol. 2026 Apr 28;26:1021. doi: 10.1186/s12870-026-08825-y (PMC13262447; doi:10.1186/s12870-026-08825-y)
Supplement: Supplementary file 2 — Additional file 2. [file 12870_2026_8825_MOESM2_ESM.docx]

**Table S1** The initial biomass, nitrogen content and δ^15^N of *Coix lacryma-jobi* L. seedlings at the experiment’s onset.

| Parameters | Plant organs | |
| --- | --- | --- |
|  | **shoot** | **Root** |
| Dry weight (g) | 0.424±0.004 | 0.216±0.008 |
| Nitrogen content (%) | 3.00±0.10 | 2.66±0.15 |
| δ^15^N (‰) | -0.1±0.0 | -1.9±0.3 |

Each value is expressed as the mean ± SE (n = 3).
